# Testing for synergistic effects of natural and anthropogenic disturbance on ecological communities at a landscape scale

**DOI:** 10.1007/s10980-024-01844-w

**Published:** 2024-02-16

**Authors:** Jed I. Lloren, J. L. McCune

**Affiliations:** Department of Biological Sciences, University of Lethbridge, 4401 University Drive, Lethbridge, AB T1K 3M4 Canada

**Keywords:** Disturbance, Non-additive effects, Recovery, Resilience, Stressors, Succession

## Abstract

**Context:**

Anthropogenic and natural disturbances may interact synergistically, magnifying their individual effects on biodiversity. However, few studies have measured responses of ecological communities to multiple stressors at landscape scales.

**Objectives:**

We use a long-term dataset to test for synergistic effects of anthropogenic and natural disturbance on plant community diversity and composition in a large protected area.

**Methods:**

We quantified changes in plant communities over two decades in 98 plots in Waterton Lakes National Park, Canada. Fifty-three plots burned in a wildfire in the interim. We modeled the effects of wildfire, proximity to trails or roads, and their interaction on changes in species richness, community composition, relative abundance of disturbance-associated species, and colonization by exotic species.

**Results:**

Interactions between wildfire and proximity to roads and trails affected all metrics except species richness. Only one interaction was synergistic: the relative abundance of disturbance-associated species following wildfire was magnified closer to recreational corridors. The other community metrics showed unexpected patterns. For example, plots with no exotic species in the baseline survey that burned in the wildfire were more likely to gain exotic species than unburned plots only when they were distant from recreational corridors.

**Conclusions:**

Our study demonstrates interactive effects of natural and anthropogenic disturbance at landscape scales within a protected area. Plant community response to wildfire was influenced by proximity to recreational corridors, sometimes in surprising ways. As the frequency and severity of anthropogenic and natural disturbances both continue to rise, documenting the prevalence and magnitude of interactions between them is key to predicting long-term effects and designing mitigation strategies.

**Supplementary Information:**

The online version contains supplementary material available at 10.1007/s10980-024-01844-w.

## Introduction

A fundamental goal of ecology is to understand how ecological communities respond to disturbance. The trajectory of change following the destruction of many or most living individuals in a community by events like wildfires, storms, insect outbreaks, or volcanic eruptions has been a preoccupation of ecologists from the earliest days of the discipline (McIntosh 1985; Meiners et al. 2015). More recently, ecologists have realized that natural disturbances are affecting ecological communities in a context of increasing human domination of the biosphere. Human activities can alter natural disturbance regimes—by suppressing wildfires, for example (Nowacki and Abrams 2008). In addition, disturbance or stressors caused by human activities can change the trajectory of recovery following natural disturbances, or even reduce the resilience of ecological communities to natural disturbances (i.e. their ability to return to their former state; Folke et al. 2004). A key challenge for ecology is to understand how anthropogenic and natural disturbances interact to influence the resilience of ecological communities (Paine et al. 1998; Turner 2010; Smart et al. 2014; Côté et al. 2016).

Many studies have examined the effects of multiple stressors on populations and communities. Effects can be additive, whereby each stressor is independent of the other (Côté et al. 2016). Alternatively, the two stressors can interact. If the interaction is synergistic, the magnitude of the effect of one stressor is amplified by the other (Côté et al. 2016), potentially leading to ‘ecological surprises’ (Paine et al. 1998). Meta-analyses of multi-stressor studies show that synergistic interactions are quite common when the response is measured at the population level, but rare at the community level (Crain et al. 2008; Côté et al. 2016). However, most studies at the community level examined changes in biomass or species richness (Orr et al. 2020). These metrics may be less responsive to disturbance because of complementarity: species that are sensitive to a disturbance or combination of disturbances decline or disappear, but those that are less sensitive increase or colonize, minimizing changes in species richness and biomass (Breitburg et al. 1998). Also, most multi-stressor studies are done at small spatial and temporal scales in the lab (e.g. most aquatic studies: Crain et al. 2008), warming chambers or mesocosms (e.g. Dieleman et al. 2012), or with small-scale experimental manipulations in nature (e.g. Micheli et al. 2016). Few multi-stressor studies have examined the response of community composition to anthropogenic and natural stressors at large spatial and temporal scales.

**Fig. 1:**
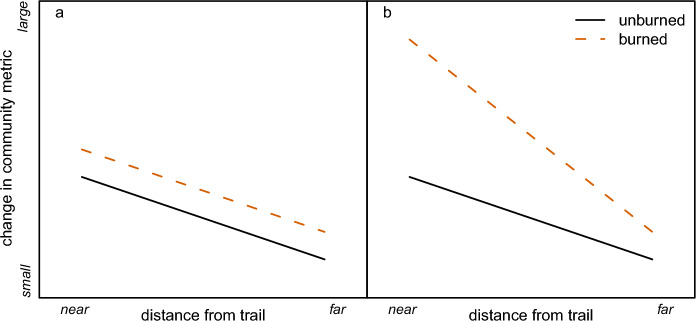
The predicted relationship between changes in plant community diversity or composition, burn status, and distance from recreational trails if wildfire and recreation are **a** additive, or **b** interact synergistically. In **a**, wildfire increases the magnitude of change in ecological communities over time relative to unburned communities consistently, regardless of the distance from a trail. In **b**, wildfire increases the magnitude of change in ecological communities over time relative to unburned communities more in areas near trails than in areas far from trails

In this study, we use data from 98 vegetation plots to test for interactions between natural and anthropogenic disturbance on changes in plant community diversity and composition in Waterton Lakes National Park in Alberta, Canada. The plots were surveyed in the early 1990s and resurveyed in 2019/2020, after about half of them were burned by a severe wildfire in 2017. We use the proximity of recreational roads and trails as a proxy for the degree of anthropogenic disturbance from recreation. We quantify changes in species richness, shifts in community composition, changes in the relative abundance of disturbance-associated species, and colonization by exotic species in each plot. We evaluate two possibilities for the effects of wildfire and recreation on these community metrics. If effects are additive, we predict that the magnitude of change in species richness and community composition will be highest in burned plots, and increase with proximity to roads or trails, but that these two drivers will not interact (Fig. 1a). If these two stressors interact synergistically, we predict that close proximity to trails or roads will magnify the effect of the wildfire on the plant community (Fig. 1b).

## Methods

### Study area

**Fig. 2:**
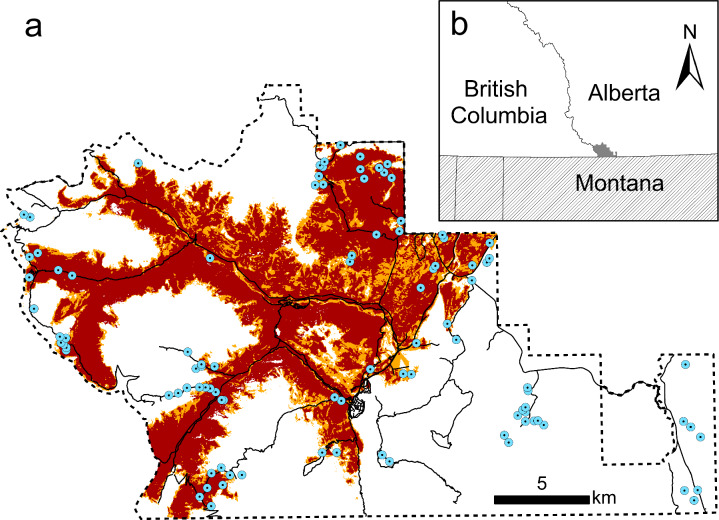
**a** Waterton Lakes National Park (WLNP) outline in black dashed line. Shading indicates the area burned by the 2017 Kenow Fire at high severity (class 5, dark red) or low to moderate severity (classes 1 through 4, orange). The points show the locations of resurveyed Ecological Land Classification (ELC) plots and the solid black lines show roads and trails. **b** The location of WLNP (grey shading) within the province of Alberta, and in relation to the nearby areas in Canada (white) and the United States (diagonal line shading)

Waterton Lakes National Park (WLNP; 49° 08′ N 113° 92′ W) is located in the southwest of the province of Alberta, Canada, in the Rocky Mountains (Fig. 2). The Park includes four ecoregions: foothills parkland, montane, subalpine, and alpine (Strong and Leggatt 1992). The foothills parkland ecoregion features fescue grasslands and aspen (*Populus tremuloides*) groves. The montane and subalpine ecoregions are characterized by coniferous forests dominated by lodgepole pine (*Pinus contorta*), with subalpine forests differentiated by the co-dominance of Engelmann spruce (*Picea engelmannii*). The alpine ecoregion vegetation is mainly composed of low shrubs.

Wildfires are the most prominent natural disturbance in WLNP. Two large lightning-ignited, non-intersecting wildfires have occurred over the past three decades: the 1998 Sofa Fire and 2017 Kenow Fire. The Sofa Fire burned about 1,500 ha of mostly coniferous forest (Buckler 2012). The Kenow Fire burned approximately 35,000 hectares including about 50% of the vegetation at WLNP (Greenaway et al. 2018; Fig. 2). Recreational use is the main anthropogenic disturbance at WLNP. In 2016, over 500,000 people visited WLNP, compared to about 350,000 visitors in 1996 (Parks Canada 2008, 2019). Recreational disturbance is primarily concentrated on roads and trails that facilitate access throughout the park. Two two-lane paved roads allow car access and bicycling; one of these roads is closed from November until May while the other stays open year-round. There are 200 km of hiking trails throughout the Park, ranging in width from 30 cm to 3 m. The busiest trails are those that allow day-hikes from road access points, while back-country trails requiring multi-day trips are less frequented. Mountain biking and horseback riding are permitted on a sub-set of designated trails only. No motorized vehicles are permitted on trails. Most visitors (80%) come to the park between May and October, which is also the peak season for plant growth.

### Data collection

From 1994–1999, Achuff et al. (2002) established 330 plots to classify the vegetation of WLNP. They surveyed 20 m × 20 m plots in forested areas, 15 m × 15 m plots in shrublands, and 10 m × 10 m plots in grasslands. Surveyors recorded the coordinates of the plot centres using a handheld Global Positioning System (GPS), identified all vascular plant species found within each plot, and estimated the percent cover of each species. In October 2017, WLNP workers used satellite imagery (30 m resolution) to map the area burned by the Kenow Fire. They used Normalized Burn Ratio (NBR) values (Key and Benson 2006) to delineate five burn severity classes based on a comparison of near-infrared and mid-infrared reflectance of vegetation pre- and post-fire. They ground-truthed the resulting map in the summer of 2018, finding high accuracy. Most of the burned area was assessed as high severity (class 5, Fig. 2).

We re-located and re-surveyed 98 of the original plots in 2019 and 2020, two to three growing seasons after the Kenow Fire. Fifty-three plots had burned in the Kenow Fire. None had burned in the earlier Sofa Fire. We chose plots to re-survey with the goal of maximizing the range of elevation, slope, vegetation type, and distance from roads or trails. We followed the sampling protocol of the original surveys, and placed a permanent marker at each plot centre. We took small samples of difficult species (e.g. sedges and grasses) back to the laboratory for identification. We carefully standardized plant species names across surveys (for details see Supporting Information, Supplemental Methods and Results).

### Analyses

We refer to the original survey period as “1994” and the re-survey years as “2019”. We evaluated the change in four community metrics for each plot. First, we measured the change in species richness by subtracting the total number of plant species present in the plot in 1994 from the number present in 2019. Second, we used the Bray–Curtis dissimilarity between each plot in 2019 and the same plot in 1994 as a measure of change in community composition (Bray and Curtis 1957). We calculated the Bray–Curtis dissimilarity based on the square-root transformed species abundance matrix. Bray–Curtis dissimilarity ranges from 0 to 1, where a value of 0 would indicate a plot that has the same species in the same relative abundances in 2019 as it did in 1994, and values closer to 1 indicate greater shifts in the presence and relative abundance of species between 1994 and 2019.

Third, we assessed plot-level changes in the abundance of disturbance-associated species. This allowed us to assess the nature of the shifts in community composition in relation to the characteristics of the species. We used habitat descriptions in two regional Floras to classify each species as disturbance-associated or not (Kuijt 1982; Moss and Packer 1994; Table S1). We categorized a species as disturbance-associated if its habitat description included terms like: burned areas, disturbed areas, clearings, roadsides, waste grounds, rockslides, gardens, and lawns. We calculated the community-weighted mean (cwm) disturbance association for each plot in each time period based on the relative abundance of each species present multiplied by 1 (disturbance-associated) or 0 (not disturbance-associated). We then subtracted the value for 1994 from the value for 2019. A positive value indicates an increase in the relative abundance of disturbance-associated species over time.

Finally, we determined whether each plot had been colonized by one or more new exotic species, or not. Most of the exotic species present in the study area are disturbance-associated (Table S2). Although the spread of invasive exotic species is an ongoing concern in the Park, the mean relative abundance of exotic species in our survey plots is very low (1994 mean: 1.03%; 2019 mean 3.09%). Therefore, we measured the colonization of each plot by new exotic species as an additional indicator of plant community change. We used the Alberta Conservation Information Management System (ACIMS) database (Kershaw 2015) to designate each species as native or exotic to Alberta. Exotic species are those that are present in the province due to direct or indirect human intervention. We then determined which plots had one or more exotic species in 2019 that were not recorded in the plot in 1994.

We used linear and generalized linear models to assess the effect of burn status (burned versus unburned), distance from the plot to the nearest road or trail, and their interaction on the four community metrics (see Table S3). We considered evaluating burn severity, but most of the 53 burned plots had burned at the highest severity (class 5; n = 36), with only 6 plots burned in severity classes 2 and 3, and none in class 1, the lowest severity. Evaluating burn status in three categories (unburned, n = 45; moderately burned, n = 17; severely burned, n = 36) resulted in very similar results (see Supporting Information). We obtained road and trail shapefiles from Parks Canada workers. They created the shapefiles based on GPS points taken in 2008 and manual corrections using aerial photographs. We used a Geographical Information System to measure the straight-line distance from the center of each plot to the nearest road or trail. Distances ranged from 2 m to 1,592 m (mean: 344 m, median: 197 m).

In each model, we also included covariates and interactions known or hypothesized to influence the response variable in question (Table S3). We included species richness in 1994 in the model for change in species richness to account for the tendency of species richness to revert to the mean over time. We included the difference in survey date between the original and re-survey in the models for change in species richness and shifts in community composition to account for the fact that seasonal timing of vegetation surveys can influence whether certain species are detected. In the model for gain versus no gain of at least one new exotic plant species over time, we included as a predictor whether the plot had at least one exotic species at the time of the original survey. The presence of one exotic species may indicate conditions favourable to other exotic species that we did not measure (e.g. soil nutrient availability).

Environmental covariates included elevation, slope, aspect, and soil drainage. We determined the elevation and aspect of each plot based on a 25 m Digital Elevation Model (Alberta Environment and Parks, Government of Alberta 2017). We calculated aspect as the “northness index” by subtracting 180 from each aspect in degrees, taking the absolute value, and dividing by 180. Plots facing north have a value of 1, south-facing plots will have a value of 0, and east or west facing plots both have a value of 0.5. Parks Canada workers used a clinometer to measure slope at each plot during the original surveys, and dug soil pits to determine the soil drainage for each plots during the 1990s surveys according to the methods in Day (1983). We collapsed soil drainage from seven categories to three (1 and 2 = well drained, 3 = moderately well drained, 4 to 7 = poorly drained) to avoid categories with few or no plots. See Supporting Information (Supplemental Methods and Results) for descriptions and justifications for the interactions we included in each model. We checked for correlations between all predictors prior to building the models. No pairwise correlation had an absolute Pearson’s correlation coefficient greater than 0.50 (Table S4).

We used linear models for the change in species richness and the change in relative abundance of disturbance associated species as all assumptions of linear models were met in each case. We used a generalized linear model with a beta distribution to model the shift in community composition over time because the Bray–Curtis dissimilarity varies from 0–1 and is not binomial (i.e. not a measure of successes out of a number of trials). We used the ‘glmmTMB’ package in R to build the beta model (Brooks et al. 2017). We re-fit the final beta model using the ‘betareg’ package to obtain a ‘pseudo R-squared’ for the minimum adequate model (Cribari-Neto and Zeileis 2010). We used a generalized linear model with a logit link to model the gain of at least one new exotic species versus no gain in new exotic species over time. Before building models we took the natural log of distance to trail to improve normality. For change in species richness, change in relative abundance of disturbance-associated species, and gain versus no gain of exotic species we used the ‘arm’ package to standardize the predictor variables by subtracting the mean and dividing by twice the standard deviation (Gelman and Su 2018). For the shift in community composition, we standardized all continuous predictors manually by subtracting the mean and dividing by the standard deviation. In the model for change in relative abundance of disturbance associated species, we removed three extreme outliers prior to building the model.

For each response variable, we first built a model with all potential predictors and interactions and used backward stepwise model selection to determine the minimum adequate model for each community metric. We ensured no spatial autocorrelation in the model residuals using spline correlograms (package ‘ncf’; Bjørnstad and Falck 2001) and ensured no model misspecification using scaled residuals (package ‘DHARMa’; Hartig 2020). We then used marginal fitting of terms (drop1 test) to determine which predictors and interactions had a significant effect while accounting for all other variables in the minimum adequate model. We report the AIC values of the model without each dropped predictor as a measure of the importance of that predictor, as well as the F-statistic and associated p-value (for linear models), or the Likelihood ratio test statistic and associated p-value (for logistic and beta-binomial models). We used partial regression plots to visualize the effect of each significant predictor while holding all other predictors constant (‘visreg’ package; Breheny and Burchett 2017). We conducted all analyses in R version 4.0.2 (R Core Team 2018). For more details, see Supporting Information.

## Results

There were 403 species (11 exotic) across all 98 plots recorded in the original surveys, and 426 species (33 exotic) in the re-surveys. The mean species richness per plot in 2019 was significantly higher than in 1994 (29 versus 26 species; Wilcoxon signed rank test, n = 98, V = 2905, p = 0.006). While plots gained species on average, the change in species richness in each plot varied widely, from a loss of 35 species to a gain of 38 species. The Bray–Curtis dissimilarity of each plot compared to the same plot in the original survey ranged from 0.23 to 0.93, with a mean of 0.64. Burned plots saw significantly greater shifts in composition than unburned plots (unburned mean dissimilarity = 0.55, burned mean dissimilarity = 0.71, Wilcoxon rank sum test, n = 98, W = 545, p < 0.001). The mean relative abundance of disturbance-associated species was 9% in 1994 (median 4%) and 20% in 2019 (median 11%). The community-weighted mean disturbance association increased significantly in burned plots (mean increase of 22%, n = 53, V = 1291, p < 0.001) and decreased very slightly in unburned plots (mean decline of 0.7%, n = 45, V = 410, p = 0.61). In the original surveys 34 plots contained one or more exotic plant species; by 2019 this had increased to 54 plots. Forty-eight plots had gained at least one new exotic species, of which twenty-three already had at least one exotic species present in 1994.

**Fig. 3:**
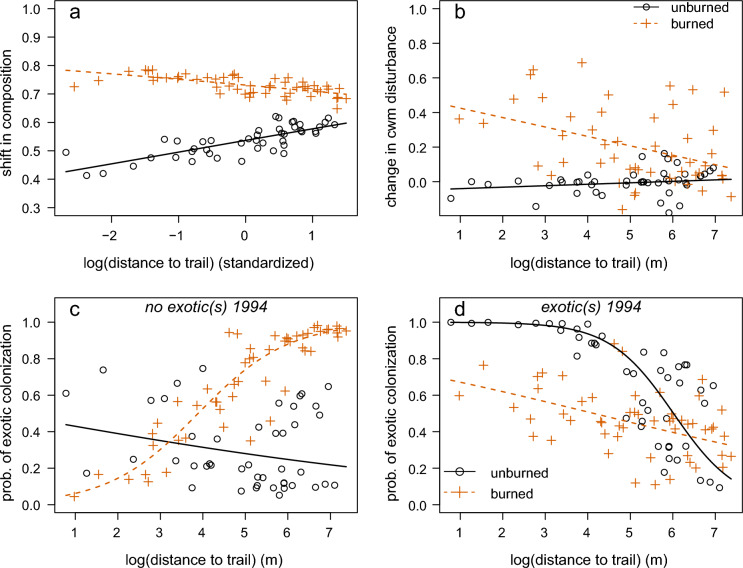
Partial regression plots showing the interaction between burn status and trail proximity on **a** change in community composition, **b** change in community-weighted mean (cwm) disturbance association, and **c**, **d** probability of colonization by one or more new exotic species in 98 vegetation plots in Waterton Lakes National Park from 1994 to 2019. In each panel, all other predictors in the model are held at their median (for continuous predictors) or the most common category (for categorical predictors), except in **c** the presence of at least one exotic in 1994 is set to ‘false’, and in **d** the presence of at least one exotic in 1994 is set to ‘true’. Confidence intervals are omitted for clarity. The legend in **b** applies to all panels

There were significant interactions between burn status and proximity to roads or trails for all community metrics except change in species richness (Fig. 3; Tables 1, 2, 3, 4; Figs. S1, S2, S3, S4). Burned plots had large shifts in composition regardless of distance from roads or trails, while unburned plots tended to see greater shifts in community composition as distance from roads or trails increased (Fig. 3a). In burned plots only, the observed increase in the relative abundance of disturbance-associated species declined with distance from roads or trails (Fig. 3b). Among plots with no exotic species present in 1994, the probability of colonization by one or more exotics increased as distance from roads or trails increased, but only for plots that were burned (Fig. 3c). In unburned plots, the probability of gaining a new exotic species declined slightly with increasing distance from roads or trails (Fig. 3c). Among plots that already had one or more exotic species present in 1994, gains of new exotic species were more likely closer to roads or trails, especially in unburned plots (Fig. 3d). The proportion of the variance in the four community metrics explained by the minimum adequate models, as measured by the R^2^ or deviance explained, was 23% for species richness and approximately 40% for the other community metrics.

**Table 1:** Summary of the linear model assessing potential predictors of change in species richness from 1994 to 2019

Response	Predictor	Coefficient	SE	df	SS	RSS	AIC^a^	F-value	p-value
Change in species richness	Intercept	2.81	1.07	1		9858.7	467.89		
	**Species richness in 1994**	−10.07	2.44	1	1864.33	11,723.1	482.87	17.02	** < 0.001**
*Adjusted R-squared: 0.23*	Elevation	−10.61	2.39	*na*	*na*	*na*	*na*	*na*	*na*
	Burn status	−3.91	2.14	*na*	*na*	*na*	*na*	*na*	*na*
	Distance to trail or road	3.87	2.21	*na*	*na*	*na*	*na*	*na*	*na*
	Burn status × distance to trail or road	6.56	4.32	1	252.42	10,111.1	468.37	2.30	0.115
	**Elevation × burn status**	−9.85	4.33	1	565.97	10,424.7	471.36	5.17	**0.019**
	Elevation × distance to trail or road	6.93	5.03	1	207.85	10,066.6	467.94	1.90	0.153

Only predictors and interactions included in the minimum adequate model (as selected by backward stepwise model selection) are included. p-values refer to the significance of each predictor after marginal fitting of terms (F-test comparing model without the predictor to the full model, using drop1). Coefficients for burn status are compared to the reference category ‘unburned’

^a^AIC of the model including all factors except the one being tested. To respect marginality, we do not drop individual predictors that are also included in an interaction (therefore *na* is shown for those predictors)

Predictors in boldface have a significant influence on the response

**Table 2:** Summary of the generalized linear model (link = beta distribution) assessing potential predictors of change in community composition (measured as the pairwise Bray–Curtis dissimilarity) from 1994 to 2019

Response	Predictor	Coefficient	SE	df	AIC^a^	LRT	p-value
Change in community composition	Intercept	0.15	0.08		−122.85		
	**Elevation**	0.23	0.06	1	−110.48	14.371	** < 0.001**
*Pseudo R-squared: 0.42*	**Northness**	0.25	0.06	1	−109.63	15.215	** < 0.001**
	Burn status	0.85	0.12	*na*	*na*	*na*	*na*
	Distance to trail or road	0.17	0.08	*na*	*na*	*na*	*na*
	**Burn status × distance to trail or road**	−0.27	0.12	1	−119.53	5.323	**0.021**

Only predictors and interactions included in the minimum adequate model (as selected by backward stepwise model selection) are included. p-values refer to the significance of each predictor after marginal fitting of terms (likelihood ratio test comparing model without the predictor to the full model, using drop1). Coefficients for burn status are compared to the reference category ‘unburned’

^a^AIC of the model including all factors except the one being tested. To respect marginality, we do not drop individual predictors that are also included in an interaction (therefore *na* is shown for those predictors)

Predictors in boldface have a significant influence on the response

**Table 3:** Summary of the linear model assessing potential predictors of change in relative abundance of disturbance-associated species from 1994 to 2019

Response	Predictor	Coefficient	SE	df	SS	RSS	AIC^a^	F-value	p-value
Change in relative abundance of disturbance-associated species	Intercept	0.11	0.16				−349.02		
*Adjusted R-squared: 0.37*	Burn status	0.21	0.03	*na*	*na*	*na*	*na*	*na*	*na*
	Distance to trail or road	−0.08	0.03	*na*	*na*	*na*	*na*	*na*	*na*
	**Burn status × distance to trail or road**	−0.20	0.06	1	0.23109	2.4472	−341.60	9.49	**0.002**

Only predictors and interactions included in the minimum adequate model (as selected by backward stepwise model selection) are included. p-values refer to the significance of each predictor after marginal fitting of terms (F-test comparing model without the predictor to the full model, using drop1). Coefficients for burn status are compared to the reference category ‘unburned’

^a^AIC of the model including all factors except the one being tested. To respect marginality, we do not drop individual predictors that are also included in an interaction (therefore *na* is shown for those predictors)

Predictors in boldface have a significant influence on the response

**Table 4:** Summary of the generalized linear model (link = beta distribution) assessing potential predictors of colonization of at least one new exotic species versus no new exotic(s) colonization from 1994 to 2019

Response	Predictor	Coefficient	SE	df	Deviance	AIC^a^	LRT	p-value
Gain of at least one new exotic species vs. not	Intercept	0.21	0.33		80.101	100.10		
	Presence/absence of exotic(s) in 1994	0.37	0.74	*na*	*na*	*na*	*na*	*na*
*Explained deviance: 41%*	Elevation	−3.12	0.91	*na*	*na*	*na*	*na*	*na*
	**Northness**	1.62	0.61	1	88.111	106.11	8.01	**0.004**
	Burn status	0.71	0.65	*na*	*na*	*na*	*na*	*na*
	Distance to trail or road	0.09	0.64	*na*	*na*	*na*	*na*	*na*
	**Pres/abs of exotic(s) in 1994 × distance to trail/road**	−3.68	1.66	1	86.080	104.08	5.98	**0.014**
	**Burn status × distance to trail/road**	3.48	1.43	1	87.610	105.61	7.51	**0.006**
	**Pres/abs of exotics(s) in 1994 × burn status**	−3.54	1.55	1	86.325	104.33	6.22	**0.013**
	**Elevation × burn status**	−4.32	1.74	1	88.007	106.01	7.90	**0.005**

Only predictors and interactions included in the minimum adequate model (as selected by backward stepwise model selection) are included. p-values refer to the significance of each predictor after marginal fitting of terms (likelihood ratio test comparing model without the predictor to the full model, using drop1). Coefficients for burn status are compared to the reference category ‘unburned’

^a^AIC of the model including all factors except the one being tested. To respect marginality, we do not drop individual predictors that are also included in an interaction (therefore *na* is shown for those predictors)

Predictors in boldface have a significant influence on the response

## Discussion

Landscape-scale studies are necessary to determine how interactions between anthropogenic and natural disturbances may influence the resilience of ecological communities (Breitburg et al. 1998; Orr et al. 2020). Previous landscape-scale studies have shown that when natural vegetation is fragmented, openings in these fragments created by natural disturbance are more likely to be colonized by generalist, disturbance-associated species, altering the trajectory of recovery (Catterall et al. 2008; Laurance and Curran 2008; Smart et al. 2014; Lloren et al. 2020). However, the degree to which recreation within protected areas influences the response of ecological communities to natural disturbance is largely unknown. While these areas are protected from habitat conversion, networks of roads and trails allow hiking and other recreational activities, and the intensity and extent of these activities are increasing due to rapidly increasing numbers of visitors (Monz et al. 2021). Trail networks are well-known conduits for disturbance-associated native and exotic species (Mount and Pickering 2009; Yang et al. 2021), and—in combination with natural disturbances that remove competition and increase nutrient levels—could facilitate colonization of these species beyond trail edges, leading to novel communities.

At WLNP, communities that burned in the wildfire had greater shifts in composition than those that did not burn. This is not surprising: the fire killed nearly all trees, and there was a dramatic increase of common fire followers, such as *Chamerion angustifolium* (fireweed). We expected that shifts in composition caused by the fire would be magnified near recreational trails, but this was not the case. Shifts in community composition were driven largely by losses of species, rather than gains (Fig. S5). Therefore, although plots closer to recreational corridors saw increases in the relative abundance of disturbance-associated species after the fire, this was not enough to magnify the large shifts in composition already caused by losses of formerly dominant species. Even unburned plots saw a great deal of turnover between 1994 and 2019, with a mean Bray–Curtis dissimilarity of 0.55. Interestingly, unburned plots had greater shifts in composition farther from trails—perhaps indicating a role for recreational corridors in maintaining shade-intolerant species. Forest expansion and densification in this region has been ongoing since at least the late 1800s (Stockdale et al. 2019), and we know this trend has continued from 1994 to 2019 in the unburned plots based on significant increases in the relative abundance of woody plants over this timeframe (Lloren 2021).

The change in relative abundance of disturbance-associated species matched our prediction for a synergistic response, although there was no effect of trail proximity without wildfire. In burned plots the increase in the relative abundance of disturbance-associated species was magnified in plots closer to trails or roads. This is consistent with the hypothesis that recreational trails increase the availability of propagules of disturbance-associated species (e.g. Wells et al. 2012; Wedegärtner et al. 2022), which are therefore able to quickly colonize burned areas near trails. Unburned plots saw relatively little change in the relative abundance of disturbance-associated species regardless of their proximity to trails or roads. This suggests that the near doubling of visitor levels in the Park over the past 25 years has not caused substantial increases in the abundance of disturbance-associated species—at least, not yet.

Exotic plant species tend to be more abundant near trails (e.g. Rew and Johnson 2010; Romme et al. 2011; Wells et al. 2012). We expected, therefore, to find a higher likelihood of colonization by exotic species in both burned and unburned plots that were closer to trails or roads, and highest where the wildfire removed the canopy and reduced competition. Instead, we found that wildfire increased the likelihood of colonization by exotics, but only in plots that did not already have at least one exotic species, and this facilitation of exotic colonization by the fire was greater in plots *farther* from trails or roads. Perhaps plots near trails with no exotics at the time of the first survey had some unmeasured property making them less favourable to exotic species, such as low productivity (e.g. Brodie et al. 2021), and therefore even after fire the probability of colonization by exotics was low. In contrast, plots farther from trails with no exotics in 1994 may have lacked them simply due to competition from established native species, and the fire removed competitors and released nutrients, allowing exotics to colonize. Plots that already had exotic species in 1994 were more likely to gain new exotics if they were closer to trails. However, this effect was reduced in burned plots, an antagonistic rather than a synergistic interaction. It is possible that many burned plots near trails were colonized by new exotic species at some point since 1994, but that in some cases these colonizers were killed by the fire—thereby lowering the probability of our surveys detecting the colonization. The unexpected nature of the interaction between wildfire and trail proximity on exotic species colonization highlights the possibility for ‘ecological surprises’ (Paine et al. 1998). The way that the interaction depends on baseline conditions shows that the nature of the response of an ecological community to multiple stressors can be contingent on other factors, including abiotic conditions that influence a community’s susceptibility to change.

We do not know whether the interactions we observed will affect the resilience of these communities: that is, their ability to return to their pre-fire state. Drivers of succession include: site conditions and history, species availability, and species performance (Meiners et al. 2015). Here, we show evidence for interactions between a natural ‘pulse’ disturbance—wildfire, which has altered site conditions—and an anthropogenic ‘press’ disturbance—recreational roads and trails, which primarily alter species availability (Smart et al. 2014). As Meiners et al. (2015) note, species availability is likely to be most important in transitional phases of succession, as is the case here, just two to three growing seasons after the wildfire. The effect of trail proximity may diminish over time. The early stages of post-wildfire succession in the Rocky Mountains tend to be unpredictable, whereas later succession proceeds predictably towards a coniferous canopy with an understory of shrubs and shade-tolerant herbs (Lyon and Stickney 1976). Over the long term, communities tend to be dominated by species that were present in the community before the fire (Doyle et al. 1998; Romme et al. 2011; Abella and Fornwalt 2015). However, some exotic species can maintain their presence over the long term (Lyon and Stickney 1976; Doyle et al. 1998). Continued monitoring of these plots will allow us to determine whether communities closer to roads or trails are less likely to return to their pre-fire composition.

We did not survey the plots immediately prior to the 2017 wildfire. Therefore, the changes we measured in the burned plots are the sum of changes that occurred since 1994 in addition to the effects of the fire, rather than changes due to the fire alone. However, the changes we observed in the unburned plots—specifically, shifts towards taller, woody species (Lloren 2021)—suggest that our estimates of the magnitude of the fire effect are likely more conservative than if we had compared post-fire communities to the immediate pre-fire state. We also acknowledge that the magnitude of the effect of distance from trails or roads likely varies with the intensity of trail or road use. Wider trails and roads with frequent vehicle traffic often support larger populations of disturbance-associated and exotic species, which may extend farther from the trail edge (e.g. Potito and Beatty 2005; Downing 2020; Chisholm and McCune 2024). We were not able to include a measure of trail use intensity in addition to trail proximity as a covariate in our models due to lack of quantitative data on usage frequency or intensity. However, we note that the intensity of trail use in WLNP in general tends to decline with increasing elevation, and we did not find interactions between proximity to trails and elevation for any of our response metrics.

Managers of protected areas that allow public access have to balance outdoor recreation with the protection of ecological communities. Recreational disturbance has well-documented ecological impacts (e.g. Wells et al. 2012; Monz et al. 2021; Wedegärtner et al. 2022). In addition, our study shows that it can interact with natural disturbance. Whether these interactions are a concern depends on management goals. While the number of species in a community did not show a synergistic response, the relative abundance of disturbance-associated species did. Documenting the prevalence of interactions between anthropogenic and natural disturbances globally, and measuring their longevity, is necessary to predict long-term effects and inform potential management strategies to minimize undesired outcomes.

## Conclusions

The potential for synergies between stressors is a popular topic of study (Côté et al. 2016; Orr et al. 2020), yet very little research has tested for synergistic effects of natural and anthropogenic stressors on community composition over large spatial and temporal scales. Using an extensive dataset covering a wide range of vegetation types within a large protected area, we found clear interactions between wildfire and proximity to recreational corridors on metrics of change in plant communities. However, there was no significant interaction effect on species richness, highlighting the importance of tracking other metrics that capture changes in community composition. Importantly, the nature of the interactive effects of wildfire and recreational disturbance were often surprising, and depended on the starting conditions of the community. Our work offers a baseline against which to compare the effects of interactions between natural and anthropogenic disturbance on communities at landscape scales in other regions and biomes. More large-scale studies investigating the response of communities to concurrent anthropogenic and natural disturbances will help us to predict the long-term effects of these interactions.

### Supplementary Information

Below is the link to the electronic supplementary material.

Supplementary file1 (PDF 859 KB)

## Data Availability

The data used to conduct the analyses are available in the Federated Research Data Repository, 10.20383/103.0844.
