# Supplementary material for: Testing for synergistic effects of natural and anthropogenic disturbance on ecological communities at a landscape scale
Source: Landsc Ecol. 2024 Feb 16;39(3):40. doi: 10.1007/s10980-024-01844-w (PMC10895711; doi:10.1007/s10980-024-01844-w)
Supplement: Supplementary file 1 — Supplementary file1 (PDF 859 KB) [file 10980_2024_1844_MOESM1_ESM.pdf]

Supporting Information. Lloren, J.I., and McCune, J.L. Testing for synergistic effects of natural and anthropogenic disturbance on ecological communities at a landscape scale

Supplementary Methods and Results

ELC Study

From 1994-1999, Achuff et al. (2002) established 330 plots to classify the vegetation of WLNP. They divided areas of the Park into mapped polygons based on landform features and selected plots in areas of relatively homogenous vegetation. They surveyed 20m x 20m plots in forested areas, 15m x 15m plots in shrublands, and 10m x 10m plots in grasslands. Crews identified all vascular plant species found within each plot and estimated the percent cover of each species in seven different height layers, which were later compressed into four layers. We re-located and re-surveyed 98 of the original plots in 2019 and 2020, two to three growing seasons after the Kenow fire. We standardized plant species names across all surveys and lumped species to the genus level where identification to species is difficult (for details see Lloren 2021). We collapsed the four vegetation layers into one by summing the estimated percent cover of each species in all four layers prior to analysis. On two occasions, we conducted surveys with P. Achuff, allowing us to ensure consistency with the original survey.

Analyses

We modeled four community metrics: change in species richness, change in community composition, change in community-weighted disturbance association, and gains of one or more new exotic species over time (Table S3). We tested for a significant effect of the interaction between proximity to the nearest road or trail (a proxy of human disturbance) and burn status

(natural disturbance) for each metric. We used the *near* function in ArcGIS (ESRI 2020) to measure the nearest straight-line distance from the centre of each plot to the nearest official road or trail. We obtained road and trail shapefiles from Parks Canada workers. They created the shapefiles based on GPS points taken in 2008 and manual corrections using aerial photographs. We considered evaluating burn severity, but most of the burned plots were burned at the highest severity (class 5; n=36), with only 6 plots burned in severity classes 2 and 3, and none in class 1, the lowest severity. Therefore, we categorized each plot's burn status as either unburned or burned. Most of the area burned in the Kenow Wildfire received a severity rating of 5. We also repeated our analyses by designating severity classes 2 through 4 as one category called 'moderate', and analyzing the predictor burn status as a 3-category predictor (unburned, moderately burned, severely burned). The results were similar to the results using burn status as a 2-category predictor, and we present them below for readers who are interested.

We also included covariates and interactions known or hypothesized to influence the response variable in question (Table S3). We included species richness in 1994 in the model for change in species richness to account for the tendency of species richness to revert to the mean over time. We included the difference in survey date between the original and re-survey in the models for change in species richness and shifts in community composition to account for the fact that seasonal timing of vegetation surveys can influence whether certain species are detected. In the model for gain versus no gain of at least one new exotic plant species over time, we included as a predictor whether the plot had at least one exotic species at the time of the original survey. The presence of one exotic species may indicate conditions favourable to other exotic species that we did not measure (e.g. productivity, Brodie et al. 2021). Environmental covariates included elevation, slope, aspect, and soil drainage. We determined the elevation and aspect of each plot

based on a 25m Digital Elevation Model (Alberta Environment and Parks, Government of Alberta 2017). We calculated the northness index by subtracting 180 from each aspect in degrees, taking the absolute value, and dividing by 180. Plots facing north have a value of 1, south-facing plots will have a value of 0, and east or west facing plots both have a value of 0.5. Parks Canada workers used a clinometer to measure slope at each plot during the original surveys, and dug soil pits to determine the soil drainage for each plots during the 1990s surveys according to the methods in Day (1983). We collapsed soil drainage from seven categories to three (1 and 2 = well drained, 3 = moderately well drained, 4 to 7 = poorly drained) to avoid categories with few or no plots. Before building the models, we checked for correlations between all predictors (Table S4).

Because trails and roads can act as vectors for seed transport and well-drained soils have been shown to be ideal for plant development, we included an interaction between soil drainage and distance to the nearest trail or road. We included the interaction between elevation and burn status because plant regeneration decreases with increasing elevation and this response is amplified by wildfire where lower elevations gain more species and higher elevations tend to experience greater losses after fire (e.g. Coop et al. 2010). We included an interaction between elevation and the distance to the nearest trail or road because lower elevations are more heavily travelled, thus the influence of trail proximity may be less evident at higher elevations. Finally, for the model of gains versus no gain of at least one new exotic species, we included two additional interactions. First, we tested for an interaction between the presence or absence of at least one exotic species in 1994 and the distance to the nearest trail or road. Plots near trails and roads (which are sources of disturbance and propagules of exotic plants) and plots already having at least one exotic species (indicating conditions amenable to exotics) may be more likely to gain

additional exotic species than plots near trails and roads but without any exotic species present. Second, we included an interaction between the presence or absence of at least one exotic species in 1994 and burn status. We expected that burned plots may have a greater probability of being colonized by exotic species over time than unburned plots only if they did not already have exotics in 1994 (thereby requiring the fire disturbance to open up space and/or release nutrients).

We used linear models for the change in species richness and the change in relative abundance of disturbance associated species as all assumptions of linear models were met in each case. We used a generalized linear model with a beta distribution to model the shift in community composition over time because the Bray-Curtis dissimilarity varies from 0-1 and is not binomial (i.e. not a measure of successes out of a number of trials). We used the 'glmmTMB' package in R to build the beta model (Brooks et al. 2017). We re-fit the final beta model using the 'betareg' package to obtain a 'pseudo R-squared' for the minimum adequate model (Cribari-Neto and Zeilis 2010). We used a generalized linear model with a logit link to model the gain of at least one new exotic species vs. no gain in new exotic species over time. Before building models we took the natural log of distance to trail to improve normality. For change in species richness, change in relative abundance of disturbance-associated species, and gain vs. no gain of exotic species we used the 'arm' package to standardize the predictor variables by subtracting the mean and dividing by twice the standard deviation (Gelman and Su 2018). For the shift in community composition, we standardized all continuous predictors manually by subtracting the mean and dividing by the standard deviation. In the model for change in relative abundance of disturbance associated species, we removed 3 extreme outliers prior to building the model. We used backward stepwise model selection ('MASS' package; Venables and Ripley 2002) to determine the minimum adequate model for each community metric.

We ensured no spatial autocorrelation in the model residuals using spline correlograms (package ‘ncf’; Bjørnstad and Falck 2001) and checked for problems with model specification (including overdispersion) using scaled residuals (package ‘DHARMa’; Hartig 2020). We used marginal fitting of terms (drop1 test) to determine which predictors and interactions had a significant effect while accounting for all other variables in the minimum adequate model. We report the AIC values of the model without each dropped predictor as a measure of the importance of that predictor, as well as the F-statistic and associated p-value (for linear models), or the Likelihood Ratio test statistic and associated p-value (for logistic and beta models). We used partial regression plots to visualize the effect of each significant predictor while holding all other predictors constant (‘visreg’ package; Breheny and Burchett 2017).

In the main manuscript text, we present the results using burn status (unburned vs. burned) as a 2-category predictor. Below, we also present the results using burn status as a 3-category predictor, where the status was unburned (n=45), moderately burned (severity class 2, 3, or 4, n=17), or severely burned (severity class 5, n=36). See Tables S5, S6, S7, S8 and Figures S6, S7, S8 and S9.

Table S1: List of disturbance-associated species, and their origin (exotic or native in Alberta).

Scientific name	Origin
Achillea millefolium	Native
Agoseris glauca	Native
Agrostis scabra	Native
Allium cernuum	Native
Alyssum alyssoides	Exotic
Antennaria racemosa	Native
Arctium minus	Exotic
Arctostaphylos uva-ursi	Native
Artemisia absinthium	Exotic
Artemisia borealis	Native
Artemisia michauxiana	Native
Astragalus robbinsii	Native
Bromus inermis	Exotic
Bromus vulgaris	Native
Chamerion angustifolium	Native
Chenopodium album	Exotic
Cicuta maculata	Native
Cirsium arvense	Exotic
Cirsium vulgare	Exotic
Descurainia sophia	Exotic
Draba aurea	Native
Dracocephalum parviflorum	Native
Dryas drummondii	Native
Epilobium ciliatum	Native
Equisetum sp.	Native
Festuca ovina	Exotic
Gaillardia aristata	Native
Galium boreale	Native
Gentianella amarella	Native
Geranium bicknellii	Native
Heracleum sphondylium	Native
Heuchera parviflora	Native
Hieracium umbellatum	Native
Lepidium densiflorum	Native
Lepidium latifolium	Exotic
Medicago lupulina	Exotic
Melilotus officinalis	Exotic
Monarda fistulosa	Native
Myosotis stricta	Exotic
Phleum pratense	Exotic

**Scientific name**	**Origin**
Poa compressa	Exotic
Potentilla argentea	Exotic
Potentilla rivalis	Native
Ranunculus acris	Exotic
Ranunculus aquatilis	Native
Ranunculus gmelinii	Native
Rosa acicularis	Native
Sisymbrium altissimum	Exotic
Taraxacum officinale	Exotic
Townsendia parryi	Native
Tragopogon dubius	Exotic
Trifolium pratense	Exotic
Trifolium repens	Exotic
Verbascum thapsus	Exotic

Table S2: List of all exotic species recorded, and whether they are disturbance-associated or not. 'NA' indicates species not treated in the floras we consulted.

**Scientific name**	**Disturbance associated?**
Agrostis stolonifera	No
Alyssum alyssoides	Yes
Arctium minus	Yes
Arenaria serpyllifolia	No
Artemisia absinthium	Yes
Bromus inermis	Yes
Carum carvi	No
Centaurea stoebe	NA
Chenopodium album	Yes
Cirsium arvense	Yes
Cirsium vulgare	Yes
Cynoglossum officinale	No
Descurainia sophia	Yes
Dianthus armeria	NA
Festuca ovina	Yes
Hypericum perforatum	NA
Lepidium latifolium	Yes
Leucanthemum vulgare	Yes
Medicago lupulina	Yes
Melilotus officinalis	Yes
Myosotis stricta	Yes
Phleum pratense	Yes
Plantago major	No
Poa compressa	Yes
Potentilla argentea	Yes
Ranunculus acris	Yes
Sisymbrium altissimum	Yes
Taraxacum officinale	Yes
Thlaspi arvense	No
Tragopogon dubius	Yes
Trifolium pratense	Yes
Trifolium repens	Yes
Verbascum thapsus	Yes

Table S3: Predictor variables and model specifications for each response variable.

Response	Predictors	Variable type (units/categories)
**Change in species richness** (species richness in 2019 MINUS species richness in 1994) model: lm	Species richness 1994	Continuous (count)
	Difference in survey date	Continuous (days)
	Elevation	Continuous (metres)
	Northness	Continuous (index)
	Slope	Continuous (degrees)
	Soil drainage	Categorical (poor, medium, well)
	Burn status	Categorical (unburned, burned)
	Distance to trail/road	Continuous (metres)
	Soil drainage x distance to trail/road	Categorical x continuous
	Burn status x distance to nearest trail or road	Categorical x continuous
	Burn status x elevation	Categorical x continuous
	Distance to trail/road x elevation	Continuous x continuous
**Change in community composition** (Bray-Curtis dissimilarity of each plot in 2019 compared to itself in 1994) model: glmm (link: beta)	Difference in survey date	Continuous (days)
	Elevation	Continuous (metres)
	Northness	Continuous (index)
	Slope	Continuous (degrees)
	Soil drainage	Categorical (poor, medium, well)
	Burn status	Categorical (unburned, burned)
	Distance to trail/road	Continuous (metres)
	Soil drainage x distance to trail/road	Categorical x continuous
	Burn status x distance to trail/road	Categorical x continuous
	Burn status x elevation	Categorical x continuous
	Distance to nearest trail or road x elevation	Continuous x continuous
**Change in relative abundance of disturbance-associated species** (community-weighted mean disturbance association in 2019 MINUS cwm disturbance association in 1994) model: lm	Elevation	Continuous (metres)
	Northness	Continuous (index)
	Slope	Continuous (degrees)
	Soil drainage	Categorical (poor, medium, well)
	Burn status	Categorical (unburned, burned)
	Distance to trail/road	Continuous (metres)
	Soil drainage x distance to trail/road	Categorical x continuous
	Burn status x distance to trail/road	Categorical x continuous
	Burn status x elevation	Categorical x continuous
	Distance to nearest trail or road x elevation	Continuous x continuous
**Gain (1) versus no gain (0) of at least one NEW exotic species** model: glm (link = logit)	Pres/abs of at least one exotic in 1994	Categorical (no or yes)
	Elevation	Continuous (metres)
	Northness	Continuous (index)
	Slope	Continuous (degrees)
	Soil drainage	Categorical (poor, medium, well)
	Burn status	Categorical (unburned, burned)
	Distance to trail/road	Continuous (metres)
	Pres/abs of exotic(s) in 94 x dist. to trail/road	Categorical x continuous
	Pres/abs of exotic(s) in 94 x burn status	Categorical x categorical
	Soil drainage x distance to trail/road	Categorical x continuous
	Burn status x distance to trail/road	Categorical x continuous
	Burn status x elevation	Categorical x continuous
	Distance to nearest trail or road x elevation	Continuous x continuous

Table S4: Pairwise Pearson correlation coefficients between all response and predictor variables. Coefficients with an absolute value greater than 0.40 are in boldface.

	Bray-Curtis dissimilarity	gain/no gain of new exotic(s)	change in cwm disturbance association	species richness in 1994	difference in date of survey	elevation	northness	slope	soil drainage	burn status	distance to trail/road	pres/abs of exotic(s) in 1994
change in species richness	-0.16	**0.43**	-0.31	-0.21	-0.15	-0.24	0.12	-0.17	-0.08	-0.17	0.06	0.09
Bray-Curtis dissimilarity	NA	0.17	**0.46**	-0.01	0.03	0.19	0.14	0.17	0.13	**0.48**	0.16	0.03
gain/no gain of new exotic(s)		NA	-0.04	0.33	-0.12	**-0.50**	0.35	-0.12	-0.04	0.12	0.09	0.27
change in cwm disturbance association			NA	-0.04	0.08	0.01	-0.04	0.15	0.05	**0.47**	-0.14	-0.14
species richness in 1994				NA	0.30	**-0.42**	0.25	0.00	-0.02	0.12	0.23	**0.48**
difference in date of survey					NA	0.06	0.30	0.04	-0.12	-0.03	0.33	0.07
elevation						NA	-0.19	**0.45**	0.25	-0.09	0.11	**-0.46**
northness							NA	-0.05	-0.18	-0.24	0.18	0.02
slope								NA	0.37	0.11	-0.02	-0.34
soil drainage									NA	0.14	-0.00	-0.04
burn status										NA	0.10	0.16
distance to trail/road											NA	0.15
pres/abs of exotic(s) in 1994												NA

Table S5: Summary of the model assessing potential predictors of change in species richness from 1994 to 2019, with burn status assessed as a categorical variables with 3 levels (unburned, moderately burned, and severely burned). Only predictors and interactions included in the minimum adequate model (as selected by backward stepwise model selection) are included. P-values refer to the significance of each predictor after marginal fitting of terms (F-test using drop1)

**Response**	**Predictor**	**Coefficient**	**SE**	**df**	**SS**	**RSS**	**AIC***	**F-value**	**p-value**
Change in species richness	intercept	5.08	1.59			10107	470.33		
	**species richness in 1994**	-10.32	2.49	1	1928.29	12036	485.44	17.17	**<0.001**
adjusted R-squared: 0.22	elevation	-5.08	3.37	*na*	*na*	*na*	*na*	*na*	*na*
	burn status (moderate)	-3.12	3.16	*na*	*na*	*na*	*na*	*na*	*na*
	burn status (severe)	-3.98	2.39	*na*	*na*	*na*	*na*	*na*	*na*
	distance to trail or road	3.61	2.29	1	278.05	10385	470.99	2.48	0.119
	**elevation x burn status (moderate)**	-5.35	5.76	2	725.80	10833	473.13	3.23	**0.044**
	**elevation x burn status (severe)**	-12.64	4.97						

*AIC of the model including all factors except the one being tested. To respect marginality, we do not drop individual predictors that are also included in an interaction (therefore *na* is shown for those predictors).

Note: coefficients for burn status are compared to the reference category ‘unburned’.

Table S6: Summary of the model assessing potential predictors of change in community composition (measured as the pairwise Bray-Curtis dissimilarity) from 1994 to 2019, with burn status assessed as a categorical variables with 3 levels (unburned, moderately burned, and severely burned). Only predictors and interactions included in the minimum adequate model (as selected by backward stepwise model selection) are included. P-values refer to the significance of each predictor after marginal fitting of terms (likelihood ratio test using drop1)

**Response**	**Predictor**	**Coefficient**	**SE**	**df**	**AIC***	**LRT**	**p-value**
Change in community composition	intercept	0.15	0.08		-129.66		
	**elevation**	0.23	0.06	1	-116.08	15.59	**<0.001**
	**northness**	0.25	0.06	1	-115.57	16.09	**<0.001**
pseudo R-squared: 0.48	burn status (moderate)	0.72	0.18	*na*	*na*	*na*	*na*
	burn status (severe)	1.01	0.13				
	distance to trail or road	0.17	0.08	*na*	*na*	*na*	*na*
	**burn status (mod) x dist. to trail or road**	-0.57	0.20	2	-125.41	8.26	**0.016**
	**burn status (sev) x dist. to trail or road**	-0.13	0.12				

*AIC of the model including all factors except the one being tested. To respect marginality, we do not drop individual predictors that are also included in an interaction (therefore *na* is shown for those predictors).

Note: coefficients for burn status are compared to the reference category ‘unburned’.

Table S7: Summary of the model assessing potential predictors of change in relative abundance of disturbance-associated species from 1994 to 2019, with burn status assessed as a categorical variables with 3 levels (unburned, moderately burned, and severely burned). Only predictors and interactions included in the minimum adequate model (as selected by backward stepwise model selection) are included. P-values refer to the significance of each predictor after marginal fitting of terms (F-test using drop1)

Response	Predictor	Coefficient	SE	df	SS	RSS	AIC*	F-value	p-value
Change in relative abundance of disturbance-associated species<br>*adjusted R-squared: 0.45*	intercept	0.02	0.06			1.76	-352.99		
	elevation	0.02	0.05	*na*	*na*	*na*	*na*	*na*	*na*
	soil drainage (moderate)	-0.01	0.07	*na*	*na*	*na*	*na*	*na*	*na*
	soil drainage (well)	-0.08	0.07						
	burn status (moderate)	0.28	0.05	*na*	*na*	*na*	*na*	*na*	*na*
	burn status (severe)	0.24	0.03						
	distance to trail or road	-0.18	0.15	*na*	*na*	*na*	*na*	*na*	*na*
	soil drainage (mod) x dist. to road/trail	0.23	0.16	2	0.108	1.87	-351.31	2.52	0.086
	soil drainage (well) x dist. to road/trail	0.35	0.17						
	**burn status (mod) x dist. to road/trail**	-0.58	0.13	2	0.439	2.20	-335.82	10.23	**<0.001**
	**burn status (sev) x dist. to road/trail**	-0.18	0.07						
	**elevation x burn status (moderate)**	0.15	0.09	2	0.141	1.899	-349.68	3.28	**0.043**
	**elevation x burn status (severe)**	-0.09	0.07						

*AIC of the model including all factors except the one being tested. To respect marginality, we do not drop individual predictors that are also included in an interaction (therefore *na* is shown for those predictors).

Note: coefficients for burn status are compared to the reference category ‘unburned’.

Table S8: Summary of the model assessing potential predictors of colonization of at least one new exotic species versus no new exotic(s) colonization from 1994 to 2019, with burn status assessed as a categorical variables with 3 levels (unburned, moderately burned, and severely burned). Only predictors and interactions included in the minimum adequate model (as selected by backward stepwise model selection) are included. P-values refer to the significance of each predictor after marginal fitting of terms (likelihood ratio test using drop1)

Response	Predictor	Coefficient	SE	df	Deviance	AIC*	LRT	p-value
Gain of at least one new	intercept	-0.38	0.39		83.90	107.90		
exotic species vs. not	presence/absence of exotic(s) in 1994	0.48	0.69	*na*	*na*	*na*	*na*	*na*
	elevation	-3.12	0.91	*na*	*na*	*na*	*na*	*na*
explained deviance: 38%	**northness**	1.61	0.62	1	91.64	113.64	7.74	**0.005**
	burn status (moderate)	-2.63	3.10	*na*	*na*	*na*	*na*	*na*
	burn status (severe)	0.94	0.66					
	distance to trail or road	-1.16	0.82	*na*	*na*	*na*	*na*	*na*
	**pres/abs of exotic(s) in 1994 x distance to trail/road**	-2.93	1.48	1	88.40	110.40	4.50	**0.033**
	**burn status (mod) x distance to trail/road**	10.16	7.76	2	90.28	110.28	6.37	**0.041**
	**burn status (sev) x distance to trail/road**	2.22	1.22					
	**elevation x burn status (moderate)**	-6.61	4.97	2	89.72	109.72	5.82	**0.054**
	**elevation x burn status (severe)**	-2.36	1.66					

*AIC of the model including all factors except the one being tested. To respect marginality, we do not drop individual predictors that are also included in an interaction (therefore *na* is shown for those predictors).

Note: coefficients for burn status are compared to the reference category ‘unburned

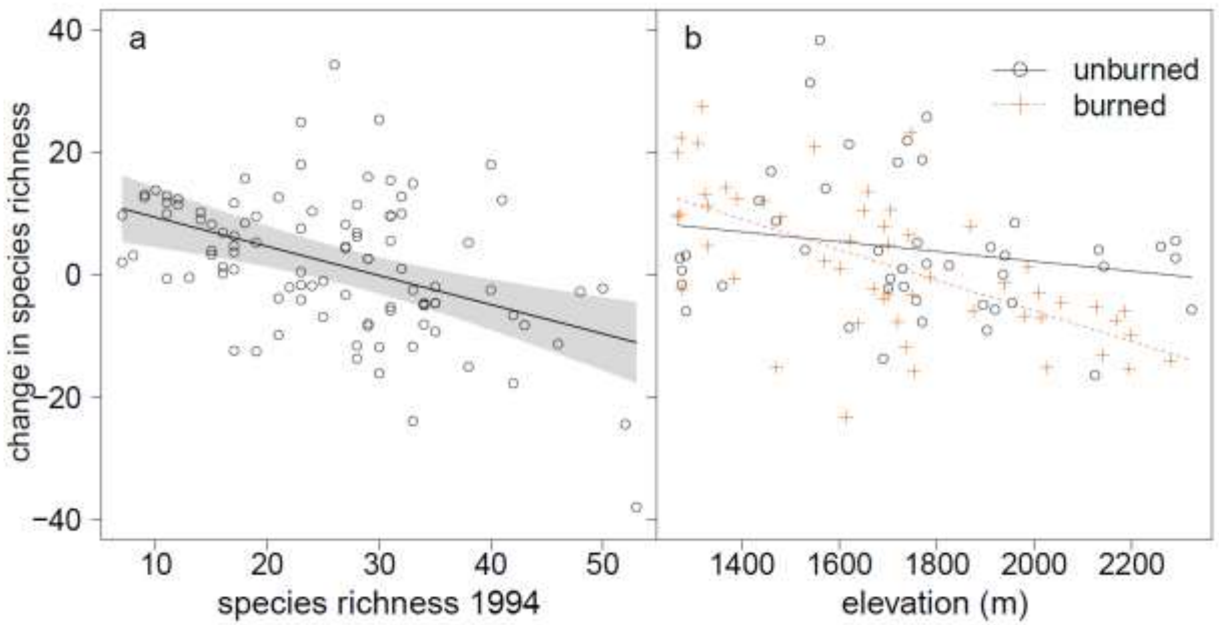

Figure S1. Partial regression plots based on the minimum adequate model for the change in species richness from 1994 to 2019 showing the effects of (a) starting species richness, and (b) the interaction between burn status and elevation. Plots are drawn based on the un-standardized model for ease of interpretation. Note that for each panel, all other variables in the model are held at the median (for continuous predictors) or the most common category (for categorical predictors). Confidence bands in (b) are omitted for clarity.

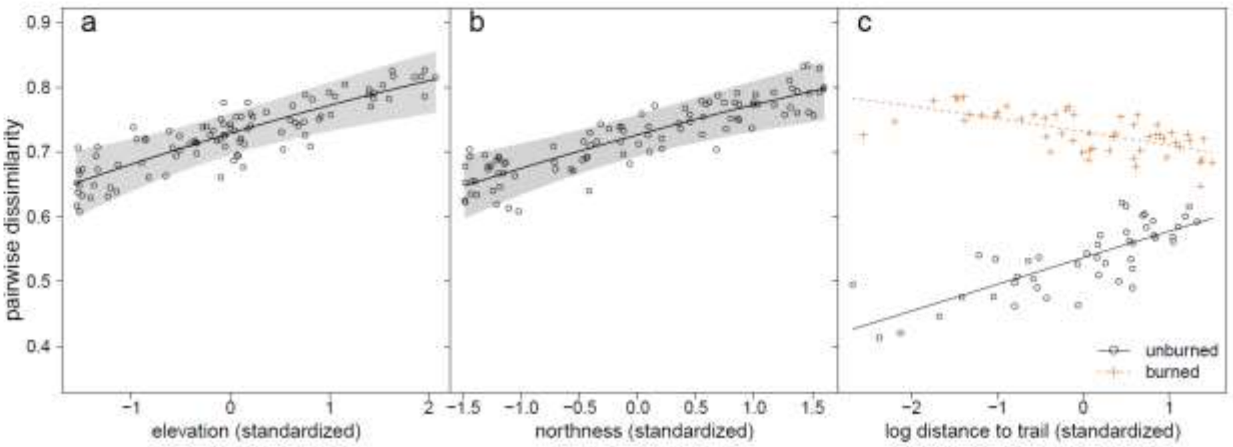

Figure S2. Partial regression plots based on the minimum adequate model for the change in community composition from 1994 to 2019 (pairwise Bray-Curtis dissimilarity) showing the effects of (a) elevation, (b) northness, and (c) the interaction between burn status and distance to the nearest road or trail. Note that for each panel, all other variables in the model are held at the median (for continuous predictors) or the most common category (for categorical predictors). Confidence bands in (c) are omitted for clarity.

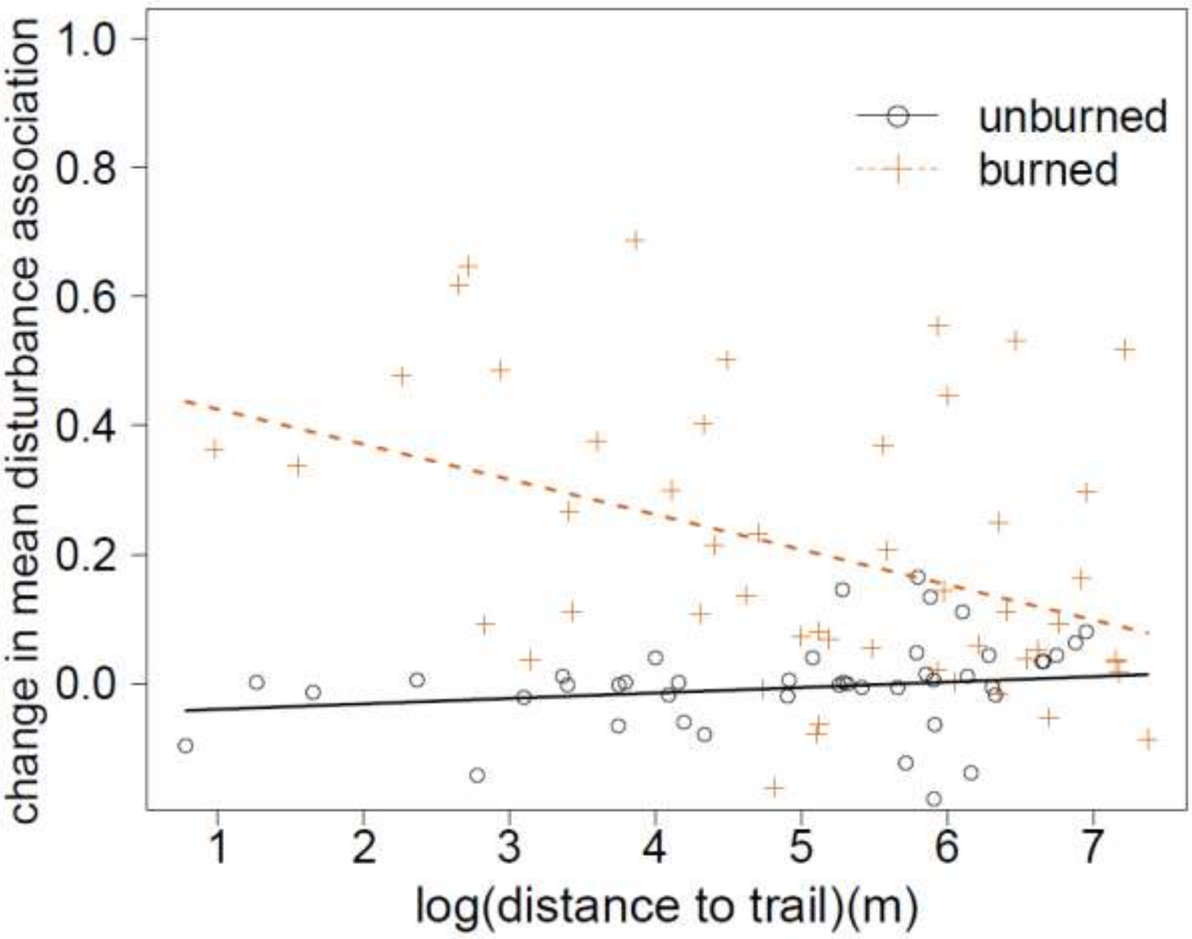

Figure S3. Partial regression plot based on the minimum adequate model for the change in community-weighted mean disturbance-association (i.e. relative abundance of disturbance-associated species) from 1994 to 2019 showing the effects of the interaction between burn status and the distance to the nearest road or trail. No other predictor variables were retained in the minimum adequate model. Confidence bands are omitted for clarity.

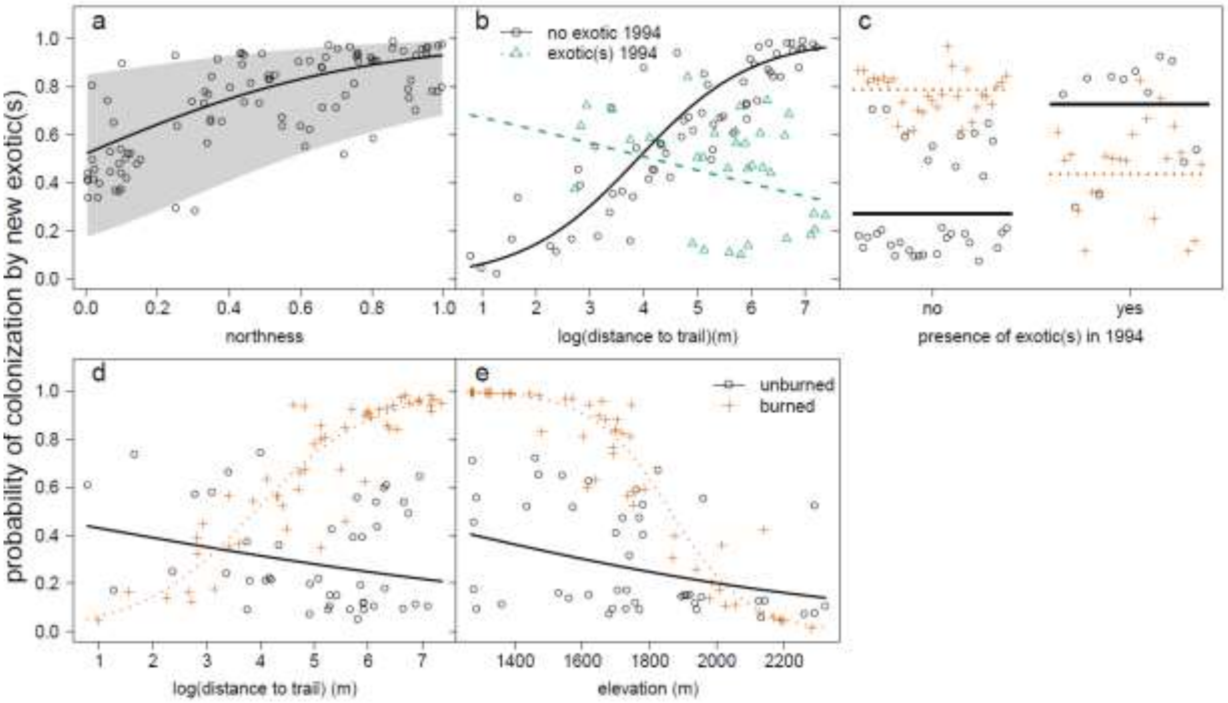

Figure S4. Partial regression plots based on the minimum adequate model for the gain versus no gain of at least one new exotic in each plot from 1994 to 2019 showing the effects of (a) northness, (b) the interaction between the presence or absence of at least one exotic in the plot in 1994 and distance to the nearest road or trail, (c) the interaction between the presence or absence of at least one exotic in the plot in 1994 and burn status, (d) the interaction between burn status and the distance to the nearest road or trail, and (e) the interaction between burn status and elevation. Note that for each panel, all other variables in the model are held at the median (for continuous predictors) or the most common category (for categorical predictors). Confidence bands in (b) through (e) are omitted for clarity. The legend in panel (e) applies to panels (c) and (d) as well.

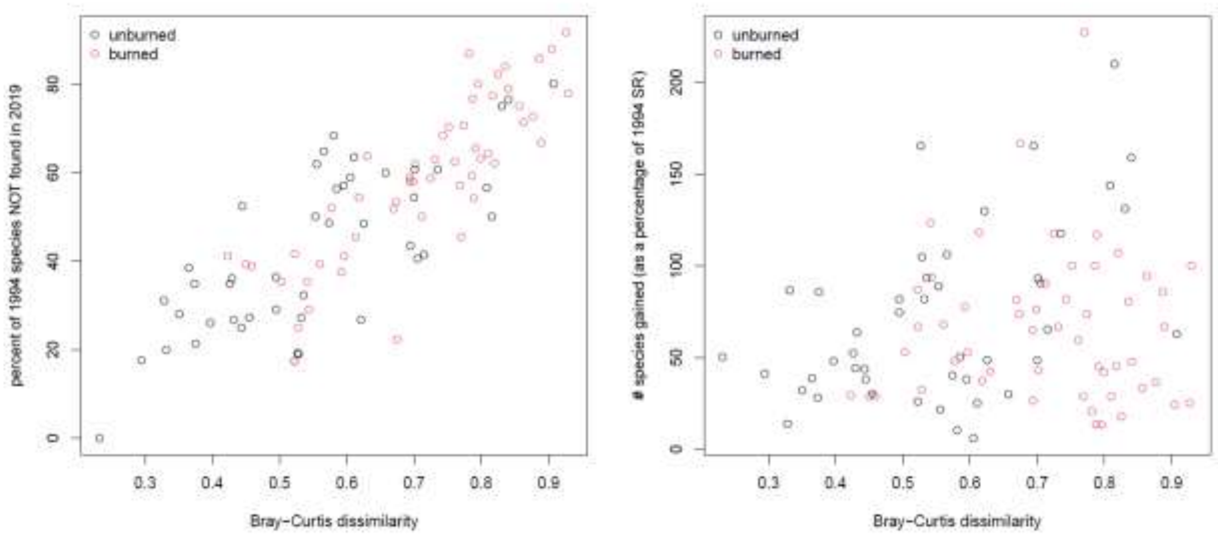

Figure S5. The percent of species present in 1994 that were not found in the 2019 resurveys is positively correlated with the pairwise Bray-Curtis dissimilarity between each plot in 2019 and the same plot in 1994 (left panel). In contrast, the number of species gained – as a percentage of baseline species richness – is not strongly correlated with Bray-Curtis dissimilarity (right panel).

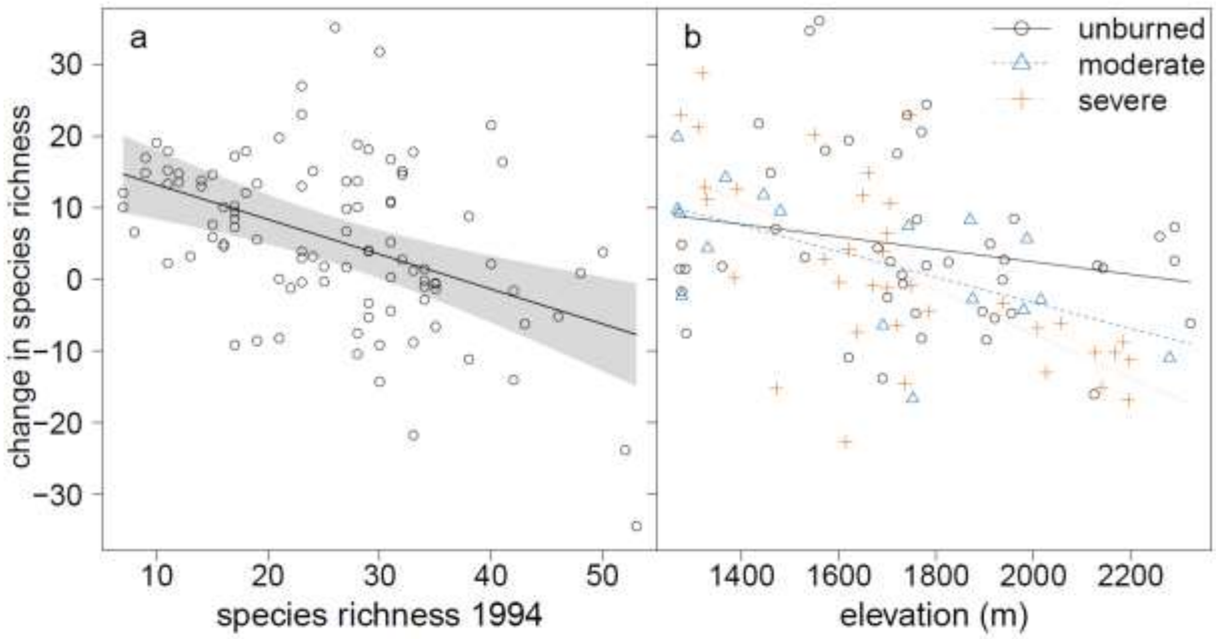

Figure S6. Partial regression plots based on the minimum adequate model for the change in species richness from 1994 to 2019 **with burn status specified in 3 categories** showing the effects of (a) starting species richness, and (b) the interaction between burn status and elevation. Plots are drawn based on the un-standardized model for ease of interpretation. Note that for each panel, all other variables in the model are held at the median (for continuous predictors) or the most common category (for categorical predictors). Confidence bands in (b) are omitted for clarity.

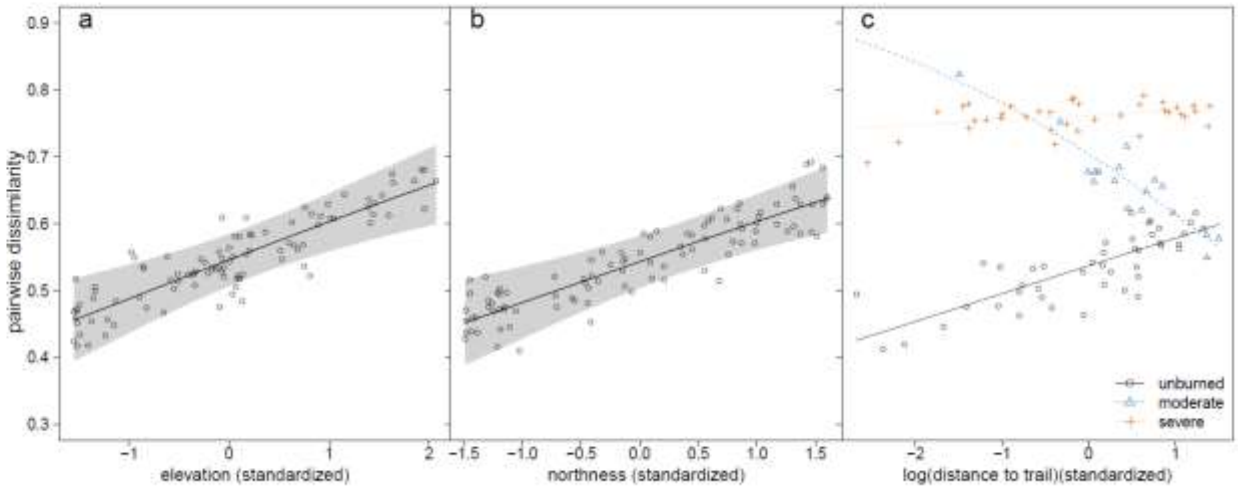

Figure S7. Partial regression plots based on the minimum adequate model for the change in community composition from 1994 to 2019 (pairwise Bray-Curtis dissimilarity) **with burn status specified in 3 categories** showing the effects of (a) elevation, (b) northness, and (c) the interaction between burn status and distance to the nearest road or trail. Note that for each panel, all other variables in the model are held at the median (for continuous predictors) or the most common category (for categorical predictors). Confidence bands in (c) are omitted for clarity.

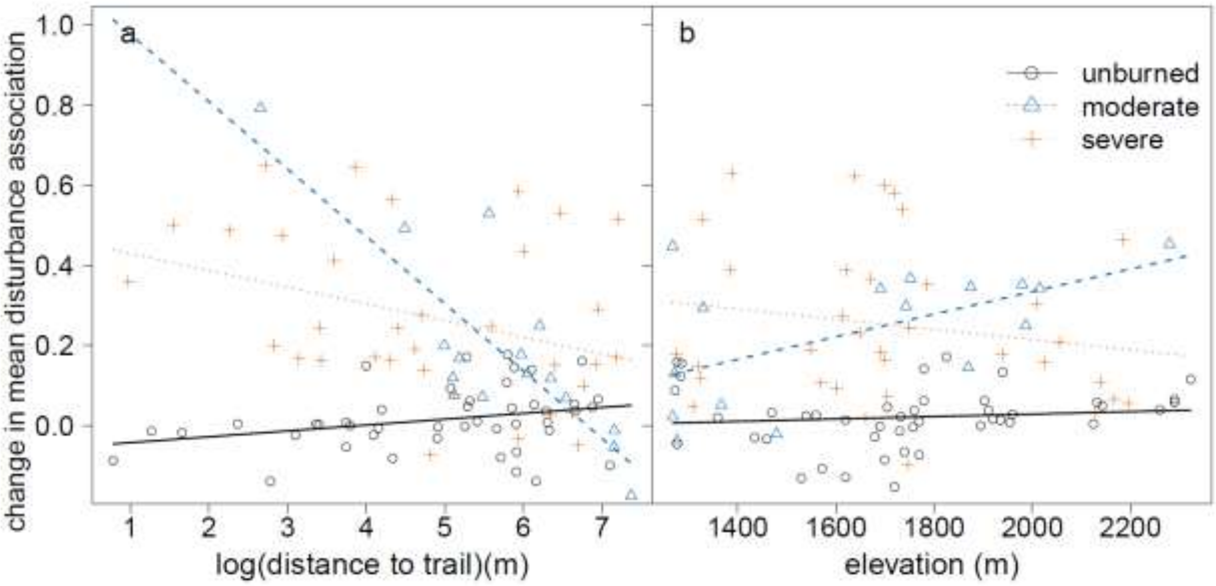

Figure S8. Partial regression plot based on the minimum adequate model for the change in community-weighted mean disturbance-association (i.e. relative abundance of disturbance-associated species) from 1994 to 2019 **with burn status specified in 3 categories** showing the effects of the interaction between burn status and the distance to the nearest road or trail and the interaction between burn status and elevation. Confidence bands are omitted for clarity.

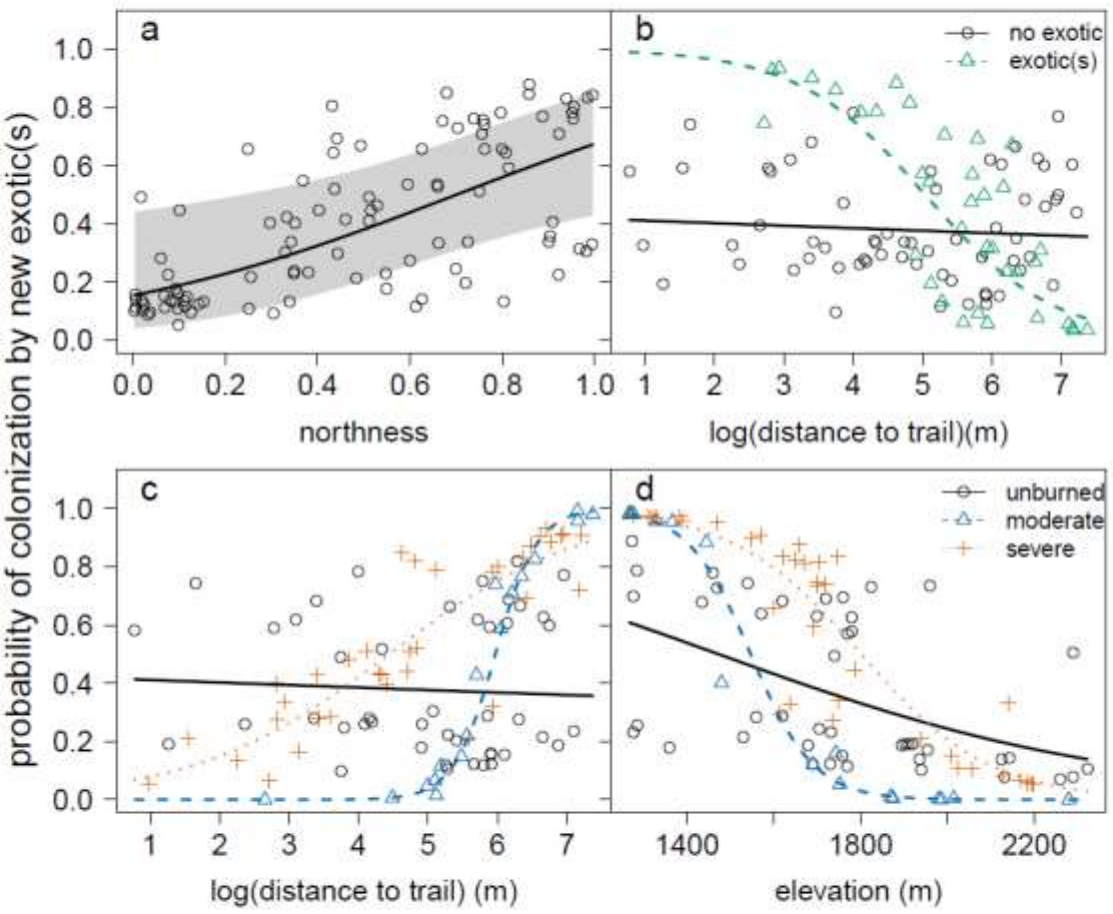

Figure S9. Partial regression plots based on the minimum adequate model for the gain versus no gain of at least one new exotic in each plot from 1994 to 2019 **with burn status specified in 3 categories** showing the effects of (a) northness, (b) the interaction between the presence or absence of at least one exotic in the plot in 1994 and distance to the nearest road or trail, (c) the interaction between burn status and the distance to the nearest road or trail, and (d) the interaction between burn status and elevation. Note that for each panel, all other variables in the model are held at the median (for continuous predictors) or the most common category (for categorical predictors). Confidence bands in (b) through (d) are omitted for clarity. The legend in panel (d) applies to panel (c) as well.

References

Achuff, P.L., McNeil, R.L., Coleman, M.L., Wallis, C., and C. Wershler. 2002. Ecological land classification of Waterton Lakes National Park, Alberta. Vol I: integrated resource description. Parks Canada, Waterton Park, Alberta.

Alberta Environment and Parks, Government of Alberta. 2017. Alberta Provincial 25 Metre Raster. Alberta Environment and Parks, Government of Alberta. Edmonton, Alberta

Bjørnstad, O.N., Falck, W. 2001. Nonparametric spatial convergence functions: Estimation and testing. Environmental and Ecological Statistics 8:53-70.

Breheny, P. and W. Burchett. 2017. Visualization of regression models using visreg. The R Journal 9:56-71.

Brodie, E. G., J. E. D. Miller, and H. D. Safford. 2021. Productivity modifies the effects of fire severity on understory diversity. Ecology 102:e03514.

Brooks, M.E., Kristensen, K., van Benthem, K.J., Magnusson, A., Berg, C.W., Nielsen, A., Skaug, H.J., Maechler, M., Bolker, B.M. 2017. glmmTMB balances speed and flexibility among packages for zero-inflated generalized linear mixed modeling. The R Journal 9:378-400.

Cribari-Neto, F., Zeileis, A. 2010. Beta regression in R. Journal of Statistical Software 34:1-24

Coop, J.D., Massatti, R.T., Schoettle, A.W. 2010. Subalpine vegetation pattern three decades after stand-replacing fire: effects of landscape context and topography on plant community composition, tree regeneration, and diversity. Journal of Vegetation Science 21:472-487.

Day, J.H. 1983. The Canada Soil Information System (CanSIS): Manual for describing soils in the field 1982 Revised. Agriculture Canada, Ottawa. 175 pp

ESRI. 2020. ArcGIS Desktop: Release 10.7.1. Redlands, CA: Environmental Systems Research Institute

Gelman, A. and Su, Y. 2018. arm: Data analysis using regression and multilevel/hierarchical models. R Package version 1.10-1

Hartig, F. 2020. DHARMa: residual diagnostics for hierarchical (multi-level/mixed) regression models. R Package version 0.3.3.0

Lloren, J. I. 2021. Quantifying plant community change at Waterton Lakes National Park over the past 25 years. MSc Thesis. University of Lethbridge, Canada.

Venables, W.N. and B.D. Ripley. 2002. Modern applied statistics with S. Springer, New York.
